# Society for Healthcare Epidemiology of America (SHEA) infectious diseases fellow infection prevention and control and healthcare epidemiology curriculum

**DOI:** 10.1017/ice.2025.85

**Published:** 2025-08

**Authors:** Elise M. Martin, Catherine Cichon, Rebecca Choudhury, Shandra R. Day, Yasaman Fatemi, Vera P. Luther, Terri Stillwell, Abby Sung

**Affiliations:** 1 Department of Medicine, Division of Infectious Diseases, University of Pittsburgh School of Medicine, Pittsburgh, PA, USA; 2 Department of Medicine, University of Colorado School of Medicine, Colorado Springs, CO, USA; 3 Mount Sinai Health System, New York, NY, USA; 4 Division of Infectious Diseases, Icahn School of Medicine at Mount Sinai, New York, NY, USA; 5 Department of Internal Medicine, Division of Infectious Diseases, The Ohio State University Wexner Medical Center, Columbus, OH, USA; 6 Division of Pediatric Infectious Diseases, Department of Pediatrics, University of Washington School of Medicine, Seattle, WA, USA; 7 Internal Medicine, Section on Infectious Diseases, Wake Forest University School of Medicine, Winston-Salem, NC, USA; 8 Division of Pediatric Infectious Diseases, Department of Pediatrics, University of Pittsburgh School of Medicine, Pittsburgh, PA, USA; 9 Division of Pediatric Infectious Diseases, UPMC Children’s Hospital of Pittsburgh, Pittsburgh, PA, USA; 10 Division of Infectious Diseases, Department of Medicine, Washington University School of Medicine, St. Louis, MO, USA

## Abstract

With the rapid expansion of the Infection Prevention Control/Healthcare Epidemiology (IPC/HE) fields over recent decades, the pivotal roles of IPC/HE in hospital regulation, quality improvement, patient safety, and healthcare finances have become increasingly apparent. Consequently, the demand for effective IPC/HE leaders has surged.^1,2^ Training in IPC/HE is essential for all infectious diseases (ID) fellows (both adult and pediatric), including those planning a career in hospital epidemiology as well as those planning to focus on general ID, transplant, HIV, etc. ID fellows, however, have historically felt ill-prepared in IPC/HE. Joiner et al’s survey highlighted this gap, revealing that only half of respondents felt adequately trained in infection control, despite half of them participating in infection control in their practice.^3^ IPC/HE fellow education is not currently standardized, and most IPC/HE training is led by individual mentors and healthcare facilities.

## General background

With the rapid expansion of the Infection Prevention Control/Healthcare Epidemiology (IPC/HE) fields over recent decades, the pivotal roles of IPC/HE in hospital regulation, quality improvement, patient safety, and healthcare finances have become increasingly apparent. Consequently, the demand for effective IPC/HE leaders has surged.^1,2^ Training in IPC/HE is essential for all infectious diseases (ID) fellows (both adult and pediatric), including those planning a career in hospital epidemiology as well as those planning to focus on general ID, transplant, HIV, etc. ID fellows, however, have historically felt ill-prepared in IPC/HE. Joiner et al’s survey highlighted this gap, revealing that only half of respondents felt adequately trained in infection control, despite half of them participating in infection control in their practice.^3^ IPC/HE fellow education is not currently standardized, and most IPC/HE training is led by individual mentors and healthcare facilities.

ID fellowship programs are expected to educate their fellows on infection prevention.^4^ ACGME Program Requirements for Infectious Diseases includes Core Competency IV.B.1.c.^5^: “Fellows must demonstrate knowledge of infection control and hospital epidemiology.”^5^ The ABIM Blueprint for the Infectious Disease Certification Exam indicates that “Infection Prevention and Control” accounts for 5% of the adult exam, and per the Pediatric Infectious Diseases Content Online, it accounts for 10% of the pediatrics exam.^6,7^ However, in a 2023 national survey of ID fellowship program directors, less than half of programs (46.3%, n = 54) reported having a formal curriculum of IPC/HE.^8^ Further, 43% of programs surveyed reported barriers to IPC/HE training, with 65% reporting that a lack of curriculum was the largest barrier to implementation. Most program directors were in favor of formal IPC/HE certification from a professional society within the standard fellowship timeframe.

We propose the Society for Healthcare Epidemiology of America (SHEA) Infection Prevention and Control/Healthcare Epidemiology (IPC/HE) Curricula to serve as a practical guide for ID training programs and fellows. Recognizing the pressing need for standardized IPC/HE fellow education, this structured curriculum is simultaneously adaptable to individual career paths and interests. The basic IPC/HE curriculum allows ID fellowship programs to ensure sufficient IPC/HE training for all fellows, regardless of career path. The advanced IPC/HE curriculum covers more advanced topics necessary for an ID physician to assume a leadership position with an IPC/HE program upon graduation. This curriculum is based on a SHEA white paper with recommended competencies for healthcare epidemiologists, current ID fellowship curricula from select institutions, expert opinion from the SHEA Education Committee, and the Infectious Diseases Society of America (IDSA) Training Program Directors’ Community of Practice.^9–13^

## Intended use

The SHEA IPC/HE Curriculum is divided into 9 categories, including (1) surveillance and reporting; (2) cluster detection, investigation, and resolution; (3) pathogen transmission and transmission interruption; (4) environment of care; (5) diagnostic stewardship; (6) occupational health; (7) emergency preparedness; (8) hospital leadership and operations; and (9) communicating infection prevention and control (IPC) work. Antimicrobial stewardship is largely excluded, since it falls outside the scope of this paper. Each category is organized into relevant topics, with each topic containing at least one learning objective (see Table 1). The objectives are categorized by pathway, either “Basic” or “Advanced” (see Table 2 for pathway requirements and a list of just the objectives in Supplementary Table 1). Twenty of the basic objectives are also marked as “core” objectives, and all fellows are expected to achieve these objectives. The basic objectives and activities are relevant for all ID fellows, regardless of long-term career path. Fellows planning a career in IPC/HE should meet a combination of basic and advanced objectives to achieve a more in-depth understanding of both IPC/HE and program leadership. Many of the objectives use introductory terms such as “recognize” or “understand,” since the goal for most fellows is to develop awareness of the objective rather than achieve proficiency. To achieve the objectives, fellows can engage in a variety of activities at their facility, such as reading and summarizing landmark articles, consulting with or shadowing local subject matter or IP experts, participating in their facility’s IPC/HE activities, and/or working on specific fellow projects. Activities are categorized only if they are advanced; otherwise, they are not labeled. Select activities are listed in Table 1, and additional ideas for activities are included in Supplementary Table 2. While these may be helpful suggestions, local adaptation based on local resources and fellow needs is recommended.

**Table 1.** The SHEA infectious diseases fellow infection prevention and control and healthcare epidemiology curriculum with objectives designated as core, basic, and advanced, including selected activities

SURVEILLANCE AND REPORTING		Core	Basic	Advanced
**National Healthcare Safety Network (NHSN) Definitions** **(CAUTI, CLABSI, CDI, MRSA, VAE, SSI)**				
**Objective 1:** Identify current NHSN definitions for the various healthcare associated infections (HAIs)		X	X	
	Review the NHSN definitions for commonly reported HAIs^18^			
	Review identifying HAIs for NHSN Surveillance^19^			
	Review Centers for Disease Control and Prevention (CDC)/NHSN Surveillance Definitions for Specific Types of Infections^20^			
**Objective 2:** Differentiate between surveillance definitions and clinical definitions for HAIs			X	
**Objective 3:** Explain advantages and disadvantages of NHSN surveillance definitions, including over capture vs under capture, LabID events, etc.				X
	Review NHSN’s modules^21^			
**HAI Performance Metrics**				
**Objective 1:** Compare the different metrics used in HAI measures (ie, rates vs Standardized Infection Ratio (SIRs))		X	X	
	Review the intro/overview on SIRs^22^			
	Review elements of SIRs calculation and interpretation within the NHSN guide^22^			
	Review the article discussing the paradoxical increases when device utilization decreases^23^			X
**Objective 2:** Identify organizations to which HAI metrics are reported, including reporting sites such as Hospital Compare, Leapfrog, etc.^24,25^				X
**Objective 3:** Describe the impact of HAI rates on insurance reimbursements, including Value-Based Purchasing				X
	Review information regarding Value-Based Purchasing (VBP) Programs^26^			
	Review the Centers for Medicare & Medicaid Services (CMS) overview of “The Hospital Value-Based Purchasing (VBP) Program”^27^			
**State and local county requirements for reporting**				
**Objective 1:** Identify HAIs that are reportable to local and state health departments, as defined by regional legislation				X

CLUSTER DETECTION, INVESTIGATION, AND RESOLUTION		Core	Basic	Advanced
**Cluster/Outbreak Investigation**				
**Objective 1:** Define an epidemiologically significant cluster and recognize differences in cluster detection thresholds among pathogens		X	X	
**Objective 2:** Describe the steps to conduct an outbreak investigation, including case definition, line list, epidemic curves, communication strategies, and public health notification			X	
	Identify and differentiate types of outbreaks based on epidemic curves^28^			
	Review the CDC’s guidance for outbreak investigations in healthcare settings^29^			
	Review the CDC’s outbreak response tool kits^30^			
	Perform a case-control or cohort study as part of a cluster investigation			X
**Objective 3:** Describe different techniques used in outbreak investigations to confirm transmission/genetic relatedness including organism identification, sensitivities, and whole genome sequencing.				X
**Objective 4:** Demonstrate effective communication to hospital leadership, risk management, clinical staff, patients/families, etc.				X
**Objective 5:** Differentiate between an outbreak and a pseudo-outbreak			X	
**Objective 6:** Describe how to approach an outbreak investigation when no clear source is identified				X
**Patient Exposure investigation**				
**Objective 1:** Explain the key elements of the patient exposure investigation, including identifying patients potentially exposed; incubation period; postexposure measures including prophylaxis, vaccination, monitoring, postdischarge isolation, hospital reporting		X	X	
**Surveillance and monitoring**				
**Objective 1:** Understand different methods for cluster detection/monitoring, including reactive (following positive clinical cultures) vs proactive assessment (active surveillance) that is used in your facility			X	
	Participate in development or monitoring of a cluster detection program			X

PATHOGEN TRANSMISSION AND TRANSMISSION INTERRUPTION		Core	Basic	Advanced
**Modes of Transmission**				
**Objective 1:** Describe ways in which patients acquire hospital-acquired infections (patient to patient, from hospital/environment, and from their own flora) and identify examples for each mode of transmission			X	
**Transmission-based precautions**				
**Objective 1:** Define and list transmission-based precautions used in healthcare		X	X	
	Review the CDC information on transmission-based precautions^31^			
	Review the CDC guideline for isolation precautions^32^			
**Objective 2**: Understand how each transmission-based precautions decreases the risk of pathogen transmission			X	
**Objective 3:** Describe methods for auditing transmission-based precautions			X	
	Perform audits of transmission-based precautions compliance of isolation rooms and identify common opportunities for improvement in your facility			
**Objective 4:** Recognize evidence-based practices that can increase compliance with transmission-based precautions				X
**Objective 5:** Appreciate differences in isolation and discontinuation practices between institutions				X
	Compare your facility’s practice for common contact pathogens (MRSA, Vancomycin-resistant *Enterococci* (VRE), Extended-spectrum beta-lactamase (ESBL)-producing *Enterobacterales*) to the CDC and other institutions, and assess for differences and alternative strategies			
**Tuberculosis (TB) prevention**				
**Objective 1:** Identify strategies used by healthcare facilities to reduce the risk of hospital-acquired TB for patients and staff			X	
	Review your facility’s process for discontinuing rule out TB isolation, including testing and clinical factors			
**Standard precautions**				
**Objective 1:** Explain how standard precautions are used to protect both staff and patients from pathogen transmission		X	X	
	CDC guidance on standard precautions^33^			
**Objective 2:** Understand the role of hand hygiene in infection prevention		X	X	
	Review World Health Organization guidelines for hand hygiene^34^			
	Review the SHEA/IDSA/Association for Professionals in Infection Control and Epidemiolog (APIC) Hand Hygiene Practice Recommendation’s list of essential practices and compare the list to your facility’s current strategies to improve hand hygiene^35^			X
	Review at least one study linking hand hygiene compliance to reducing HAIs			
	Perform audits of hand hygiene compliance within your facility with an infection preventionist and discuss a possible plan for improvement			X
**Objective 3:** Appreciate the basics of injection safety			X	
	Review CDC guidance on safe injection practices^36^			
**Objective 4:** Appreciate the basics of respiratory precautions			X	
	Review the CDC’s guidance on respiratory etiquette^37^			
**Special populations**				
**Objective 1:** Describe additional strategies used to reduce transmission of pathogens in special populations or specialized locations [bone marrow transplant (BMT), Solid organ transplant, Burn units, dialysis units, neonatal/pediatric intensive care unit (NICU/PICU), Ambulatory, labor and delivery (L&D), long term care, operating rooms (OR), and resource limited settings]				X
**Prevention strategies for CAUTI, CLABSI, CDI, MRSA, multidrug-resistant organisms (MDRO), SSI, etc. including bundles**				
**Objective 1:** Recognize effective strategies to reduce HAIs and/or device associated infections		X	X	
	Perform device audits focused on reducing one or more of these HAIs in your facility			X
	Participate in a quality improvement (QI) project aimed at addressing one of these HAIs			X
**Objective 2:** Describe effective strategies to reduce CAUTI			X	
	SHEA Compendium of Strategies to prevent catheter-associated urinary tract infections^38^			
**Objective 3:** Describe effective strategies to reduce CLABSI			X	
	SHEA Compendium of Strategies to prevent central line–associated bloodstream infections^39^			
**Objective 4:** Describe effective strategies to reduce *C. difficile*			X	
	SHEA Compendium of Strategies to prevent *C. difficile*^40^			
**Objective 5:** Describe effective strategies to reduce MRSA			X	
	SHEA Compendium of Strategies to prevent MRSA^41^			
**Objective 6:** Describe effective strategies to reduce SSI			X	
	SHEA Compendium of Strategies to prevent surgical site infections^42^			
**Objective 7:** Describe effective strategies to reduce VAE			X	
	SHEA Compendium of Strategies to prevent ventilator associated infections^43^			

ENVIRONMENT OF CARE		Core	Basic	Advanced
**Construction**				
**Objective 1:** Recognize pathogens associated with specific water and construction activities (Aspergillus and molds, waterborne pathogens)		X	X	
	Identify common environmental reservoirs for mold and bacterial pathogens^44^			
**Objective 2:** Identify construction related activities associated with HAIs			X	
**Objective 3:** Describe mitigation strategies for construction-associated HAIs				X
**Objective 4:** Define the purpose and general elements of an Infection Control Risk Assessment				X
	Define the elements of an Infection Control Risk Assessment (ICRA)^45^			
**Water Management**				
**Objective 1:** Identify reservoirs for common waterborne pathogens (Legionella, Pseudomonas, other Gram negatives, nontuberculous mycobacteria (NTM)		X	X	
	Review modes of transmission of waterborne infections^44^			
	Identify common reservoirs for Legionella, Pseudomonas, other Gram-negative bacteria, and NTM in healthcare facilities ^44^			
**Objective 2:** Describe strategies to interrupt the transmission of water borne pathogens, including having a water management plan			X	
**Objective 3:** Review the potential infectious risks associated with bathroom fixtures, such as toilet and sinks				X
**Objective 4:** Recognize unique infection prevention requirements and strategies in hemodialysis			X	
	Review CDC guidelines for water monitoring in dialysis facilities^44^			
	Review CMS requirements for water monitoring in dialysis facilities^46^			
**Air Quality**				
**Objective 1:** Explain the role of air handling and pressure differentials to reduce the risk of pathogen transmission			X	
**Objective 2:** Compare the air handling and pressure requirements for different areas of the healthcare facility				X
**Environmental Cleaning, Disinfection, and Sterilization**				
**Objective 1:** Explain the role of environmental and reusable medical device cleaning in transmission disruption			X	
**Objective 2:** Describe how to select an appropriate cleaning agent for a device (sporicidal agents, indications for use)			X	
**Objective 3:** Discuss the role of ultraviolet (UV) disinfection and other no touch cleaning strategies, including indications, benefits, and limitations				X
**Objective 4:** Compare methods of low-level disinfection, high-level disinfection, and sterilization for reusable medical devices		X	X	
	Review Spaulding classification system for disinfection of medical devices^47^			
	Review the CDC’s recommendations for endoscope reprocessing programs^48^			
	Review methods and challenges associated with inactivation of high consequence pathogens (transmissible spongiform encephalopathies/Creutzfeldt-Jakob disease (CJD), Ebola)			X
**Objective 5:** Review benefits and limitations of common methods to assess the effectiveness of cleaning (direct practice observation, fluorescent markers, adenosine triphosphate (ATP) bioluminescence testing, environmental cultures, etc.)				X

DIAGNOSTIC STEWARDSHIP		Core	Basic	Advanced
**Test selection, test characteristics, and impact on HAIs**				
**Objective 1:** Define diagnostic stewardship and explain connection to hospital epidemiology		X	X	
	Review the SHEA position paper on diagnostic stewardship^49^			
**Objective 2:** Illustrate principles of diagnostic stewardship in infection prevention using examples, such as CDI, blood, and urine cultures, etc.		X	X	
	Apply diagnostic stewardship principles to an existing, new, or potential project within your hospital’s IP program			X
**Objective 3:** Discuss the connections between diagnostic and antimicrobial stewardship in the context of hospital epidemiology/infection prevention (eg, *C. difficile*, urine culturing, etc.)				X
**Antimicrobial stewardship**				
**Objective 1:** Illustrate the connections between diagnostic and antimicrobial stewardship in the context of hospital epidemiology/infection prevention (eg, *C. difficile*, urine culturing, etc.)			X	
**Microbiology lab**				
**Objective 1:** Recognize the relationship between the microbiology laboratory and infection prevention (blood cultures, MDROs, urine cultures, CDI)			X	

OCCUPATIONAL HEALTH		Core	Basic	Advanced
**Staff vaccination**				
**Objective 1:** Identify core vaccinations recommended for healthcare personnel and describe their importance for infection prevention		X	X	
	Review the Advisory Committee on Immunization Practices (ACIP) recommendations for vaccination of healthcare provider (HCP)^50^			
	Review Hepatitis B vaccination requirements under the Occupational Safety and Health Administration (OSHA) Bloodborne Pathogen Standard^51^			
**Objective 2:** Describe the impact of HCP influenza vaccination on influenza-like illness and mortality in long-term care and acute care hospitals			X	
**Objective 3:** Describe strategies to improve vaccination rates among HCP				X
	Review Joint Commission Strategies for Improving Healthcare Personnel Influenza Vaccination Rates^52^			
**Presenteeism**				
**Objective 1:** Define presenteeism and describe its potential impact on HAIs			X	
**Objective 2:** Describe strategies to mitigate presenteeism			X	
	Discuss your facility’s challenges with presenteeism with respiratory viruses with IP or occupational medicine			X
**Healthcare worker exposure investigations**				
**Objective 1:** Describe principles of evaluating and managing exposures, including bloodborne and non-bloodborne pathogens		X	X	
	Review “Occupational Health Update: Approach to Evaluation of Health Care Personnel and Preexposure Prophylaxis”^53^			
**Objective 2:** Identify which body fluids and exposure mechanisms are considered high risk			X	
**Objective 3:** Identify and provide management recommendations for infections in which postexposure prophylaxis is indicated			X	
	Given a clinical case scenario, provide recommendations on whether postexposure prophylaxis is indicated (if possible, participate in a postexposure investigation)			
**Objective 4:** Distinguish the various postexposure work restrictions for infected or exposed asymptomatic HCP (eg, *Neisseria meningitidis*, Varicella zoster virus (VZV), measles, Influenza, norovirus, pertussis)				X
**TB screening and latent TB infection (LTBI) in healthcare workers**				
**Objective 1:** Interpret the results of pre-placement TB screening and provide treatment recommendations			X	
	Given a clinical case scenario, interpret HCP LTBI screening results and provide recommendations (if possible, participate in evaluation of a positive TB screening result)			
	Review the American College of Occupational and Environmental Medicine (ACOEM) and National Tuberculosis Controllers Association (NTCA) Joint Task Force recommendations for screening, testing, and treating for TB in US healthcare personnel^54^			
**Objective 2:** Describe what qualifies as an exposure TB in the healthcare setting and management/follow-up testing			X	
**Objective 3:** Describe methods for managing borderline interferon-gamma release assay (IGRA) in HCP				X

EMERGENCY PREPAREDNESS		Core	Basic	Advanced
**Emerging pathogens**				
**Objective 1:** Define emerging pathogens, including bioterrorism-related and re-emerging infectious diseases		X	X	
	Review National Institute of Allergy and Infectious Diseases (NIAID’s) list of emerging and re-emerging pathogens^55^			
**Objective 2:** Describe syndrome-based isolation/control measures for emerging infectious disease disasters in which the agent is not yet identified				X
	Review APIC’s Guide to Infection Prevention in Emergency Medical Services, Section 7: “Bioterrorism and Infectious Disease Emergency Preparedness”^56^			X
**Hospital Incident Command**				
**Objective 1:** Recognize the role and importance of the Incident Command System (ICS) and Hospital Incident Command System (HICS)				X
	Review the National Incident Management System (NIMS) course^57^			
	Review SHEA’s guidance on “Outbreak Response and Incident Management”^58^			
**Collaboration with state and local public health**				
**Objective 1:** Describe the purpose and scope of the CDC’s Health Alert Network (HAN) messaging system		X	X	
	Review the most recent updates from the HAN^59^			
**Objective 2:** Identify community workers requiring infection prevention education during an infectious disease disaster			X	
	Review APIC’s “Ambulatory Care During Disasters”^60^			
**Objective 3:** Describe policy development in response to infection-related events				X
	Review the CDC’s public health emergency response guide for state, local, and Tribal health departments^61^			
	Assist in policy and response plan development and evaluation for infection-related events (real or simulated), such as bioterrorism or pandemic respiratory pathogens			X
**Principles of pandemic preparedness**				
**Objective 1:** Describe the basics of developing a pandemic preparedness plan			X	
	Review the CDC’s resources for developing national, state, or local pandemic influenza preparedness plans^62^			
**Objective 2:** Recognize the importance of identifying and triaging potentially contagious individuals upon entering a facility, including ambulatory and community facilities			X	
	Review APIC’s “Ambulatory Care During Disasters”^63^			
**Objective 3:** Recognize the CMS Emergency Preparedness regulations and requirements to participate in the Medicare or Medicaid program				X
	Complete the CMS Emergency Preparedness Basic Training^64^			
**Objective 4:** Identify methods to reduce healthcare personnel absenteeism during an infectious disease disaster, including: prioritizing select healthcare personnel vaccination, offering prophylaxis and vaccination to personnel family members, such as Emergency Medical Services (EMS), volunteers, etc.				X

HOSPITAL LEADERSHIP AND OPERATIONS		Core	Basic	Advanced
**Hospital administration structure**				
**Objective 1:** Describe the structure of your institution’s IPC/HE program, including how it fits in the institutional organizational chart			X	
**Policy creation**				
**Objective 1:** Recognize how IP policies are created and modified with new data			X	
**Return on investment and paying for IP work**				
**Objective 1:** Recognize the importance of making a financial case for IP			X	
	Review how to make a making a business case for infection prevention^65^			
	Review CDC’s “Creating a Business Case for Infection Prevention”^66^			
**Objective 2:** List the outcomes that can be measured to estimate the cost of an HAI, such as: number of bed-days lost to a case of HAI, length of stay, hospital charges, and mortality				X
**Objective 3:** Apply return on investment (ROI) principles to an existing or potential IP/HE issue				X
**Quality improvement**				
**Objective 1:** Summarize a quality improvement framework (eg, Lean principles, plan do study act, Six Sigma) and apply it to a potential or existing IP problem		X	X	
**Objective 2:** Interpret and display HAI metric data, such as run charts and data dashboards			X	
**Objective 3:** Interpret HAI metric data using statistical process control (SPC) charts				X
**Objective 4:** Recognize the role of the SQUIRE guidelines in publishing QI work				X

COMMUNICATING IP WORK		Core	Basic	Advanced
**Publishing IP work**				
**Objective 1:** Describe the benefits and challenges of publishing infection prevention research			X	
**Objective 2:** Participate in infection prevention research with the goal of manuscript publication or presentation (oral or poster) at a local, regional, or national meeting				X
**Educating on IP topics**				
**Objective 1:** Understand the basics of creating and delivering an education session for trainees or hospital staff on an IP topic		X	X	
	Review methods and tools for effective delivery of education, such as didactic lectures, small-group case-based discussions, one-on-one instruction, simulation, unit-level training, role-play, hands-on skill training, and computer-based modules			
	Use presentation tools to develop effective educational materials targeting specific healthcare personnel, such as trainees, clinical staff, nonclinical staff, etc., and if feasible, provide that education			X
**Internal and external communication**				
**Objective 1:** List available tools for communicating with patients and staff and examine the benefits and challenges of these common tools, such as SBAR (Situation-Background-Assessment-Recommendation), debriefing, videoconferencing, digital shared file storage, and smartphone chat groups			X	
	Review the CDC’s Communication and Collaboration, section on “Possible Areas of Collaboration for Occupational Infection Prevention and Control Services”^67^			X
**Data visualization**				
**Objective 1:** Recognize the importance of data visualization strategies in improving data communication.				X
	Review the CDC’s “Health Communication Playbook” sections regarding images and graphics^68^			
	Review ICHE primer on data visualization^69^			
	Using the CDC NHSN data for your institution and a visualization software (such as Tableau), create a graphics display for frontline staff			
**Social media**				
**Objective 1:** Identify how common social media tools can be used in infection prevention communication			X	
	Review the CDC’s “The Health Communicator’s Social Media Toolkit”^70^			
	Review the article “Use of “Social Media”—an Option for Spreading Awareness in Infection Prevention”^71^			X
	Choose a social media communication strategy and create a table reviewing the inputs, activities, outputs, and short- and long-term outcomes for that strategy			X
**Objective 2:** Discuss the risks associated with social media use				X
**Media training**				
**Objective 1:** Recognize basic media communication skills and strategies for effective IP communication.		X	X	
	Review the CDC’s “Health Communication Playbook,” including communication strategies, consumer communication, media and press communication, and professional communication^68^			
	Meet with local public affairs team to discuss key effectors of effective media communication			X

CAUTI: catheter-associated urinary tract infection, CLABSI: central line–associated bloodstream infection, CDI: *Clostridioides difficile* infection, MRSA: Methicillin-resistant *Staphylococcus aureus*, VAE: Ventilator-associated event, SSI: Surgical site infection

**Table 2.** Required activities for completion and certificate for of both the basic and advanced pathways

Required Activities for Pathway Completion/Certificate		Total Items
**Basic**		
	Completion of 50% of all Basic, including the 20 “Core” objectives	31
	*Note: can substitute another objective from the same category, at the discretion of your program’s leadership, if unable to satisfy one or more of the core objectives	
**Advanced**		
	Completion of 80% basic objectives^62^	50
	Completion of at least 25% of advanced objectives^32^	8
	Scholarly project in infection prevention	1

Each ID fellowship program can incorporate the curriculum to meet its program’s needs. This document may supplement key areas of an existing basic curriculum, or it can be incorporated into programs without any existing structured IPC/HE training program. While this curriculum is meant to be relatively comprehensive, fellows and fellowship programs will have a variety of mechanisms by which to achieve the learning objectives. Ideally, program leadership and/or local IPC/HE leaders should guide each fellow in choosing one or more topics from each category of highest local priority, emphasizing meeting at least one objective through the suggested activities or local facility opportunities. Programs can use this list as a checklist to ensure a broad range of key IPC/HE topics are incorporated into each fellow’s training.

This education can also be supplemented by and combined with existing infection prevention education programs, such as the SHEA/Centers for Disease Control and Prevention Training Certification in Healthcare Epidemiology, the SHEA Primer on Healthcare Epidemiology, Infection Control, and Antimicrobial Stewardship (online ID fellows’ course), and the Annual Fellows’ Course in Healthcare Epidemiology, Infection Prevention, & Antimicrobial Stewardship (in-person course).^14–16^ The curriculum in this paper is designed to offer a more comprehensive approach and serve as a framework for longitudinal training. It can be implemented either as a several-week rotation for all fellows or as an extended program during the second and/or third year of fellowship, particularly for those aspiring to careers in infection prevention and hospital epidemiology. While this curriculum reviews collaboration with public health, fellows interested in advanced public health training could consider further collaboration with their local public health department or the Epidemic Intelligence Service.^17^

To be considered to have received adequate training in IPC/HE, this committee recommends that a fellow aiming to complete basic training (those not pursuing a career in IPC/HE) should complete at least 50% of all basic objectives, including the 20 core objectives. Those interested in advanced training in IPC/HE should complete at least 80% of all basic objectives, 25% percent of all advanced objectives, and complete a scholarly project, such as a research project or quality improvement project in IPC, with the goal of sharing it locally or nationally at a scientific meeting or manuscript publication. Local ID programs should assist ID fellows with opportunities to meet the recommended training and track their progress/completion.

The SHEA Education Committee is currently working on a certificate program as part of the 2025 work plan.

## Supporting information

Martin et al. supplementary material 1 Martin et al. supplementary material

Martin et al. supplementary material 2 Martin et al. supplementary material

## Supplementary material

The supplementary material for this article can be found at https://doi.org/10.1017/ice.2025.85.
