# Supplementary material for: Society for Healthcare Epidemiology of America (SHEA) infectious diseases fellow infection prevention and control and healthcare epidemiology curriculum
Source: Infect Control Hosp Epidemiol. 2025 Jun 3;46(8):773–82. doi: 10.1017/ice.2025.85 (PMC12483618; doi:10.1017/ice.2025.85)
Supplement: Martin et al. supplementary material 1 — Martin et al. supplementary material [file S0899823X25000856sup001.docx]

**Supplementary Table 2: SHEA ID fellow IPC/HE curriculum with expanded activity suggestions.**

| **SURVEILLANCE AND REPORTING** | | **Core** | **Basic** | **Advanced** |
| --- | --- | --- | --- | --- |
|  |  |  |  |  |
| **NHSN Definitions (CAUTI, CLABSI, CDI, MRSA, VAE, SSI)** | | | | |
| **Objective 1:** Identify current NHSN definitions for the various HAIs | | X | X |  |
|  | [Review the National Healthcare Safety Network (NHSN) definitions for commonly reported healthcare associated infections](https://www.cdc.gov/nhsn/index.html) |  |  |  |
|  | [Review Identifying Healthcare-associated Infections (HAI) for NHSN Surveillance](https://www.cdc.gov/nhsn/PDFs/pscManual/2PSC_IdentifyingHAIs_NHSNcurrent.pdf) |  |  |  |
|  | [Review CDC/NHSN Surveillance Definitions for Specific Types of Infections](https://www.cdc.gov/nhsn/pdfs/pscmanual/17pscnosinfdef_current.pdf) |  |  |  |
| **Objective 2:** Differentiate between surveillance definitions and clinical definitions for HAIs | |  | X |  |
|  | Meet with infection preventionist(s) to review the definition of a recent HAI and why it met the definition, highlighting the differences between surveillance and clinical definitions |  |  |  |
| **Objective 3:** Explain advantages and disadvantages of NHSN surveillance definitions, including over capture vs under capture, LabID events, etc | |  |  | X |
|  | [Review NHSN's modules](https://www.cdc.gov/nhsn/psc/index.html) |  |  |  |
|  |  |  |  |  |
| **HAI Performance Metrics** | | | | |
| **Objective 1:** Compare the different metrics used in HAI measures (i.e. rates vs Standardized Infection Ratio (SIRS) | | X | X |  |
|  | [Review the intro/overview on SIRs](https://urlisolation.com/browser?clickId=E9BDB37E-DF55-460C-8970-2CB800F9E5BD&traceToken=1709306708%3Bseachildrens_1_hosted%3Bhttps%3A%2Fsheaonline.sharepoint.com&url=https%3A%2F%2Fwww.cdc.gov%2Fnhsn%2Fpdfs%2Fps-analysis-resources%2Fnhsn-sir-guide.pdf) |  |  |  |
|  | [Review elements of SIRs calculation and interpretation within the NHSN guide](https://www.cdc.gov/nhsn/pdfs/ps-analysis-resources/nhsn-sir-guide.pdf) |  |  |  |
|  | [Review the article discussing the paradoxical increases when device utilization decreases](https://www.cambridge.org/core/journals/infection-control-and-hospital-epidemiology/article/abs/reporting-catheterassociated-urinary-tract-infections-denominator-matters/EA1748CE46270B4804D7C1E19FA8264A) |  |  | X |
| **Objective 2:** Identify organizations to which HAI metrics are reported, including reporting sites such as Hospital Compare, Leapfrog, etc. | |  |  | X |
|  | Review your healthcare system's data on reporting sites such as Hospital Compare, Leapfrog, etc. |  |  |  |
| **Objective 3:** Describe the impact of HAI rates on insurance reimbursements, including Value Based Purchasing | |  |  | X |
|  | [Review information regarding Value-Based Purchasing (VBP) Programs](https://www.ncbi.nlm.nih.gov/pmc/articles/PMC5161317/) |  |  |  |
|  | [Review the CMS overview of "The Hospital Value-Based Purchasing (VBP) Program"](https://www.cms.gov/medicare/quality/value-based-programs/hospital-purchasing#:~:text=The%20Hospital%20VBP%20Program%20rewards%20acute%20care%20hospitals,based%20on%20the%20quality%20of%20care%20they%20deliver.) |  |  |  |
|  | Meet with local infection prevention team to review your hospital's current value based purchasing program to understand targets and implications on reimbursement |  |  |  |
|  |  |  |  |  |
| **State and local county requirements for reporting** | | | | |
| **Objective 1:** Identify HAIs that are reportable to local and state health departments, as defined by regional legislation | |  |  | X |
|  | Meet with local infection preventionists to review local and state reporting requirements for your health system |  |  |  |
|  | Meet with infection preventionists to review regional legislation related to HAI reporting |  |  |  |
|  |  |  |  |  |
| **CLUSTER DETECTION, INVESTIGATION AND RESOLUTION** | | **Core** | **Basic** | **Advanced** |
| **Staff** |  |  |  |  |
| **Cluster/Outbreak Investigation** | | | | |
| **Objective 1:** Define an epidemiologically significant cluster and recognize differences in cluster detection thresholds among pathogens | | X | X |  |
| **Objective 2:** Describe the steps to conduct an outbreak investigation, including case definition, line list, epidemic curves, communication strategies, and public health notification | |  | X |  |
|  | Review the steps of an outbreak investigation |  |  |  |
|  | Participate in a local outbreak investigation |  |  |  |
|  | Develop a case definition and line list for a possible or actual, local outbreak |  |  |  |
|  | Identify and differentiate types of outbreaks based on epidemic curves |  |  |  |
|  | Review the CDC's guidance for outbreak investigations in outbreak settings |  |  |  |
|  | Review the CDC's outbreak response tool kits |  |  |  |
|  | Perform a case-control or cohort study as part of a cluster investigation |  |  | X |
| **Objective 3:** Describe different techniques used in outbreak investigations to confirm transmission/genetic relatedness including organism identification, sensitivities, and whole genome sequencing. | |  |  | X |
|  | Review different techniques used in outbreak investigations to confirm transmission/genetic relatedness including organism identification, sensitivities, and whole genome sequencing. |  |  |  |
| **Objective 4:** Demonstrate effective communication to hospital leadership, risk management, clinical staff, patients/families, etc | |  |  | X |
| **Objective 5:** Differentiate between an outbreak and a pseudo-outbreak | |  | X |  |
|  | Review a possible a pseudo-outbreak, including possible causes, with your local IP team |  |  |  |
| **Objective 6:** Describe how to approach an outbreak investigation when no clear source is identified | |  |  | X |
|  |  |  |  |  |
| **Patient Exposure investigation** | | | | |
| **Objective 1:** Explain the key elements of the patient exposure investigation including identifying patients potentially exposed; incubation period; post-exposure measures including prophylaxis, vaccination, monitoring, post discharge isolation, hospital reporting | | X | X |  |
|  | Review a recent facility exposure with the IP team to learn how to identify patients and providers potentially exposed |  |  |  |
|  | Review a recent facility exposure with the IP team and review the key elements of the exposure investigation including identifying patients and providers potentially exposed; incubation period; post-exposure measures including prophylaxis, vaccination, monitoring, furlough |  |  |  |
|  |  |  |  |  |
| **Surveillance and monitoring** | | | | |
| **Objective 1:** Understand different methods for cluster detection/monitoring, including reactive (following positive clinical cultures) vs proactive assessment (active surveillance) that is used in your facility | |  | X |  |
|  | Review one example of reactive (following positive clinical cultures) vs proactive assessment (active surveillance) that is used in your facility |  |  |  |
|  | Describe different situations where reactive vs proactive surveillance may be preferred |  |  |  |
|  | Participate in development or monitoring of a cluster detection program |  |  | X |
|  |  |  |  |  |
| **PATHOGEN TRANSMISSION AND TRANSMISSION INTERRUPTION** | | **Core** | **Basic** | **Advanced** |
|  |  |  |  |  |
| **Modes of Transmission** | | | | |
| **Objective 1:** Describe ways in which patients acquire hospital-acquired infections (patient to patient, from hospital/environment, & from their own flora) and identify examples for each mode of transmission | |  | X |  |
|  | Define the modes of transmission within healthcare settings and an example of each mode of transmission – including: (1) Between patients – direct contact, indirect contact (healthcare workers, fomites), respiratory (droplet, airborne), contamination of the environment – fecal oral, contact, etc (2) Hospital/environment – indirectly from other patients, from air handling, construction, water, food (contaminated food served) (3) From the patient themselves – infections from their own gut, fecal oral, fecal contamination of a wound, etc |  |  |  |
|  |  |  |  |  |
| **Transmission-based precautions** | | | | |
| **Objective 1:** Define and list transmission-based precautions (TBP) used in healthcare | | X | X |  |
|  | [Review the CDC information on transmission-based precautions](https://www.cdc.gov/infection-control/hcp/basics/transmission-based-precautions.html?CDC_AAref_Val=https://www.cdc.gov/infectioncontrol/basics/transmission-based-precautions.html) |  |  |  |
|  | [Review the CDC guideline for isolation precautions](https://www.cdc.gov/infection-control/media/pdfs/guideline-isolation-h.pdf?CDC_AAref_Val=https://www.cdc.gov/infectioncontrol/pdf/guidelines/Isolation-guidelines-H.pdf) |  |  |  |
| **Objective 2**: Understand how each transmission-based precautions decreases the risk of pathogen transmission | |  | X |  |
|  | Review the elements of contact isolation and how each element decreases the risk of contact transmission |  |  |  |
|  | Review the elements of droplet isolation and how each elements decreases the risk of droplet transmission |  |  |  |
|  | Review the elements of airborne isolation and how each elements decreases the risk of airborne transmission |  |  |  |
|  | Compare droplet and airborne isolation |  |  |  |
|  | Understand the role of aerosol generating procedures in pathogen transmission |  |  |  |
|  | Review your facility's process for discontinuing isolation precautions |  |  |  |
| **Objective 3:** Describe methods for auditing transmission-based precautions | |  | X |  |
|  | Review your facility's process for auditing compliance with transmission-based precautions |  |  |  |
|  | Perform audits for transmission-based precautions compliance of isolation rooms and identify common opportunities for improvement in your facility |  |  |  |
| **Objective 4:** Recognize evidence-based practices that can increase compliance with transmission-based precautions | |  |  | X |
|  | Develop a plan to increase compliance with personal protective equipment (PPE) on a unit or for a particular TBP type |  |  |  |
|  | Review an article on a quality improvement intervention to improve transmission-based precautions compliance |  |  |  |
| **Objective 5:** Appreciate differences in isolation and discontinuation practices between institutions | |  |  | X |
|  | Compare your facility's practice for common contact pathogens [Methicillin-resistant *Staphylococcus aureus* (MRSA), Vancomycin-resistant *Enterococci* (VRE), Extended-spectrum beta-lactamase (ESBL)-producing *Enterobacterales*] to the CDC and other institutions, and assess for differences and alternative strategies |  |  |  |
|  |  |  |  |  |
| **Tuberculosis prevention** | | | | |
| **Objective 1:** Identify strategies used by healthcare facilities to reduce the risk of hospital acquired tuberculosis (TB) for patients and staff | |  | X |  |
|  | Review your facility's TB risk assessment |  |  |  |
|  | Review your facility's process for discontinuing rule out TB isolation, including testing and clinical factors |  |  |  |
|  |  |  |  |  |
| **Standard precautions** | | | | |
| **Objective 1:** Explain how standard precautions are used to protect both staff and patients from pathogen transmission | | X | X |  |
|  | [Review the CDC guidance on standard precautions, including examples of standard precautions commonly employed by healthcare facilities (31)](https://www.cdc.gov/infectioncontrol/basics/standard-precautions.html) |  |  |  |
| **Objective 2:** Understand the role of hand hygiene in infection prevention | | X | X |  |
|  | [Review World health Organization guidelines for hand hygiene](https://www.who.int/teams/integrated-health-services/infection-prevention-control/hand-hygiene) |  |  |  |
|  | [Review the SHEA/IDSA/APIC Hand Hygiene Practice Recommendation's list of essential practices and compare the list to your facility’s current strategies to improve hand hygiene](https://www.cambridge.org/core/journals/infection-control-and-hospital-epidemiology/article/sheaidsaapic-practice-recommendation-strategies-to-prevent-healthcareassociated-infections-through-hand-hygiene-2022-update/FCD05235C79DC57F0E7F54D7EC314C2C) |  |  | X |
|  | Review your facility’s hand hygiene processes or program |  |  |  |
|  | Review at least one study linking hand hygiene compliance to reducing healthcare associated infections |  |  |  |
|  | Perform audits of hand hygiene compliance within your facility with an infection preventionist and discuss a possible plan for improvement |  |  | X |
|  | Develop a plan for improving hand hygiene in a specific clinical area |  |  | X |
| **Objective 3:** Appreciate the basics of injection safety | |  | X |  |
|  | [Review CDC guidance on safe injection practices](https://www.cdc.gov/injectionsafety/index.html) |  |  |  |
|  | Review your facility's education for staff on safe injection practices |  |  |  |
| **Objective 4:** Appreciate the basics of respiratory precautions | |  | X |  |
|  | [Review the CDC's guidance on respiratory etiquette](https://www.cdc.gov/infectioncontrol/guidelines/viral/prevent-viral.html) |  |  |  |
|  |  |  |  |  |
| **Special populations** | | | | |
| **Objective 1:** Describe additional strategies used to reduce transmission of pathogens in special populations or specialized locations [bone marrow transplant (BMT), Solid organ transplant, Burn units, dialysis units, neonatal/pediatric intensive care unit (NICU/PICU), Ambulatory, labor and delivery (L&D), long term care, operating rooms (OR), and resource limited settings] | |  |  | X |
|  | Review following list and select one special population in your facility to compare and identify unique IP practices that reduce the risk of infection in this location and why they are effective: BMT, Solid organ transplant, Burn units, dialysis units, NICU/PICU, Ambulatory, L&D, long term care, ORs, and resource limited settings |  |  | X |
|  |  |  |  |  |
| **Prevention strategies for CAUTI, CLABSI, CDI, MRSA, MDRO, SSI, etc including bundles** | | | | |
| **Objective 1:** Recognize effective strategies to reduce HAIs and/or device associated infections | | X | X |  |
|  | Attend a committee meeting focused on addressing one of these HAIs in your facility |  |  |  |
|  | Perform device audits focused on reducing one or more of these HAIs in your facility |  |  | X |
|  | Participate in a quality improvement (QI) project aimed at addressing one of these HAIs |  |  | X |
| **Objective 2:** Describe effective strategies to reduce catheter-associated Urinary Tract Infection (CAUTI) | |  | X |  |
|  | [SHEA Compendium of Strategies to prevent catheter associated urinary tract infections](https://www.cambridge.org/core/journals/infection-control-and-hospital-epidemiology/article/strategies-to-prevent-catheterassociated-urinary-tract-infections-in-acutecare-hospitals-2022-update/7A56FE9DABD0A9C670D728AD16F9FC48) |  |  |  |
|  | Meet with your facility's IP and review current CAUTI rates and ongoing projects to reduce CAUTI locally |  |  |  |
|  | Compare and contrast institutional policies with evidence-based practices in SHEA Compendium of Strategies to prevent catheter associated urinary tract infections |  |  | X |
| **Objective 3:** Describe effective strategies to reduce Central Line-associated Bloodstream Infection (CLABSI) | |  | X |  |
|  | [SHEA Compendium of Strategies to prevent central line–associated bloodstream infections](https://www.cambridge.org/core/journals/infection-control-and-hospital-epidemiology/article/strategies-to-prevent-ventilatorassociated-pneumonia-ventilatorassociated-events-and-nonventilator-hospitalacquired-pneumonia-in-acutecare-hospitals-2022-update/A2124BA9B088027AE30BE46C28887084) |  |  |  |
|  | Meet with your facility's IP and review current CLABSI rates and ongoing projects to reduce CLABSI locally |  |  |  |
|  | Compare and contrast institutional policies with evidence-based practices in SHEA Compendium of Strategies to prevent central line–associated bloodstream infections |  |  | X |
| **Objective 4:** Describe effective strategies to reduce C. difficile | |  | X |  |
|  | [SHEA Compendium of Strategies to prevent C. difficile](https://www.cambridge.org/core/journals/infection-control-and-hospital-epidemiology/article/strategies-to-prevent-ventilatorassociated-pneumonia-ventilatorassociated-events-and-nonventilator-hospitalacquired-pneumonia-in-acutecare-hospitals-2022-update/A2124BA9B088027AE30BE46C28887084) |  |  |  |
|  | Meet with your facility's IP and review ongoing projects to reduce C. difficile infections locally |  |  |  |
|  | Compare and contrast institutional policies with evidence-based practices in SHEA Compendium of Strategies to prevent C. difficile |  |  | X |
| **Objective 5:** Describe effective strategies to reduce MRSA | |  | X |  |
|  | [SHEA Compendium of Strategies to prevent MRSA](https://www.cambridge.org/core/journals/infection-control-and-hospital-epidemiology/article/sheaidsaapic-practice-recommendation-strategies-to-prevent-methicillinresistant-staphylococcus-aureus-transmission-and-infection-in-acutecare-hospitals-2022-update/5DB835D2E13F7E813A8A2FD7CB8386BD) |  |  |  |
|  | Meet with your facility's IP and review ongoing projects to reduce MRSA infections locally |  |  |  |
|  | Compare and contrast institutional policies with evidence-based practices in SHEA Compendium of Strategies to prevent MRSA |  |  | X |
| **Objective 6:** Describe effective strategies to reduce SSI | |  | X |  |
|  | [SHEA Compendium of Strategies to prevent surgical site infections](https://www.cambridge.org/core/journals/infection-control-and-hospital-epidemiology/article/strategies-to-prevent-surgical-site-infections-in-acutecare-hospitals-2022-update/2F824B9ADD6066B29F89C8A2A127A9DC) |  |  |  |
|  | Meet with your facility's IP and review ongoing projects to reduce surgical site infections locally |  |  |  |
|  | Compare and contrast institutional policies with evidence-based practices in SHEA Compendium of Strategies to prevent surgical site infections |  |  | X |
| **Objective 7:** Describe effective strategies to reduce VAE | |  | X |  |
|  | [SHEA Compendium of Strategies to prevent ventilator associated infections](https://www.cambridge.org/core/journals/infection-control-and-hospital-epidemiology/article/strategies-to-prevent-ventilatorassociated-pneumonia-ventilatorassociated-events-and-nonventilator-hospitalacquired-pneumonia-in-acutecare-hospitals-2022-update/A2124BA9B088027AE30BE46C28887084) |  |  |  |
|  | Meet with your facility's IP and review ongoing projects to reduce ventilator associated events locally |  |  |  |
|  | Compare and contrast institutional policies with evidence-based practices in SHEA Compendium of Strategies to prevent ventilator associated infections |  |  | X |
|  |  |  |  |  |
| **ENVIRONMENT OF CARE** | | **Core** | **Basic** | **Advanced** |
|  |  |  |  |  |
| **Construction** | | | | |
| **Objective 1:** Recognize pathogens associated with specific water and construction activities (Aspergillus and molds, waterborne pathogens) | | X | X |  |
|  | [Identify common environmental reservoirs for mold and bacterial pathogens](https://www.cdc.gov/infection-control/media/pdfs/Guideline-Environmental-H.pdf) |  |  |  |
| **Objective 2:** Identify construction related activities associated with HAIs | |  | X |  |
|  | Identify high risk construction activities for transmitting mold and waterborne infections |  |  |  |
| **Objective 3:** Describe mitigation strategies for construction-associated HAIs | |  |  | X |
|  | Review mitigation strategies for construction-associated HAIs used at your facility |  |  |  |
| **Objective 4:** Define the purpose and general elements of an Infection Control Risk Assessment | |  |  | X |
|  | [Define the elements of an Infection Control Risk Assessment (ICRA)](https://www.ashe.org/icra2) |  |  |  |
|  | Participate in an ICRA for a construction project at your facility |  |  | X |
|  |  |  |  |  |
| **Water Management** | | | | |
| **Objective 1:** Identify reservoirs for common waterborne pathogens (*Legionella*, *Pseudomonas*, other Gram negatives, mycobacteria/NTM) | | X | X |  |
|  | [Review modes of transmission of waterborne infections](https://www.cdc.gov/infection-control/hcp/environmental-control/water.html?CDC_AAref_Val=https://www.cdc.gov/infectioncontrol/guidelines/environmental/background/water.html) |  |  |  |
|  | [Identify common reservoirs for Legionella, Pseudomonas, other Gram-negative bacteria, and NTM in HCF](https://www.cdc.gov/infection-control/media/pdfs/Guideline-Environmental-H.pdf) |  |  |  |
|  | Review elements of water systems in healthcare facilities that contribute to infectious risk and strategies for mitigation |  |  |  |
|  | Define outbreak vs. pseudo-outbreak in the context of waterborne pathogens |  |  |  |
|  | Participate in a waterborne pseudo-outbreak investigation at your facility |  |  | X |
| **Objective 2:** Describe strategies to interrupt the transmission of water borne pathogens, including having a water management plan | |  | X |  |
|  | Attend a local water safety committee meeting |  |  |  |
| **Objective 3:** Review the potential infectious risks associated with bathroom fixtures, such as toilet and sinks | |  |  | X |
|  | Review mitigation strategies for bathroom fixture associated infections |  |  | X |
|  | Review outbreak investigations related to sink-associated infections (at your facility or a published case series) |  |  | X |
| **Objective 4:** Recognize unique infection prevention requirements and strategies in hemodialysis | |  | X |  |
|  | [Review CDC guidelines for water monitoring in dialysis facilities](https://www.cdc.gov/infection-control/hcp/environmental-control/water.html#cdc_generic_section_3-3-water-systems-in-health-care-facilities) |  |  |  |
|  | [Review CMS requirements for water monitoring in dialysis facilities](https://www.cms.gov/regulations-and-guidance/legislation/cfcsandcops/downloads/esrdfinalrule0415.pdf) |  |  |  |
|  | Join facility Infection Prevention team during audit of hemodialysis facilities |  |  | X |
|  |  |  |  |  |
| **Air Quality** | | | | |
| **Objective 1:** Explain the role of air handling and pressure differentials to reduce the risk of pathogen transmission | |  | X |  |
|  | Define negative pressure, positive pressure, and neutral pressure and indications for each |  |  |  |
|  | Define the five methods of air filtration |  |  | X |
|  | Discuss indications for HEPA filtration with your facility's Infection Prevention team |  |  | X |
|  | [Describe the principles of ultraviolet germicidal irradiation (UVGI), benefits, and limitations (72)](https://www.cdc.gov/niosh/docs/2009-105/pdfs/2009-105.pdf?id=10.26616/NIOSHPUB2009105) |  |  | X |
| **Objective 2:** Compare the air handling and pressure requirements for different areas of the healthcare facility | |  |  | X |
|  | Review air handling/exchange requirements for different areas of your healthcare facility |  |  | X |
|  |  |  |  |  |
| **Environmental Cleaning, Disinfection, and Sterilization** | | | | |
| **Objective 1:** Explain the role of environmental and reusable medical device cleaning in transmission disruption | |  | X |  |
|  | Join your facility's Infection Prevention team during an environmental cleaning audit |  |  |  |
|  | Participate in an exposure investigation related to improper processing of reusable medical devices |  |  | X |
| **Objective 2:** Describe how to select an appropriate cleaning agent for a device (sporicidal agents, indications for use) | |  | X |  |
|  | Identify commonly used hospital-grade disinfectants (i.e. peroxide, bleach, quaternary ammonia compounds, etc) |  |  |  |
|  | Discuss indications for use of sporicidal agents |  |  |  |
| **Objective 3:** Discuss the role of UV disinfection and other no touch cleaning strategies, including indications, benefits, and limitations | |  |  | X |
|  | Read an article on indications for and potential benefits of UV disinfection |  |  |  |
|  | Review your facility's use of UV disinfection or other no touch cleaning methods |  |  |  |
| **Objective 4:** Compare methods of low-level disinfection, high-level disinfection, and sterilization for reusable medical devices | | X | X |  |
|  | [Review Spaulding classification system for disinfection of medical devices](https://www.cdc.gov/infection-control/hcp/disinfection-sterilization/rational-approach.html?CDC_AAref_Val=https://www.cdc.gov/infectioncontrol/guidelines/disinfection/rational-approach.html) |  |  |  |
|  | [Review the CDC's recommendations for endoscope reprocessing programs](https://www.cdc.gov/hicpac/media/pdfs/essential-elements-508.pdf) |  |  |  |
|  | Review methods of high-level disinfection |  |  |  |
|  | Compare advantages and disadvantages of agents used for HLD |  |  | X |
|  | Review methods of sterilization |  |  |  |
|  | Visit your facility's sterile processing department along with the Infection Prevention team |  |  | X |
|  | Review methods and challenges associated with inactivation of high consequence pathogens (transmissible spongiform encephalopathies/CJD, Ebola) |  |  | X |
|  | Review your facility’s protocols for reprocessing and monitoring endoscopes and bronchoscopes |  |  |  |
|  | Shadow sterilization technicians during the reprocessing process |  |  | X |
| **Objective 5:** Review benefits and limitations of common methods to assess the effectiveness of cleaning (direct practice observation, fluorescent markers, ATP bioluminescence testing, environmental cultures, etc.) | |  |  | X |
|  |  |  |  |  |
| **DIAGNOSTIC STEWARDSHIP** | | **Core** | **Basic** | **Advanced** |
|  |  |  |  |  |
| **Test selection, test characteristics, and impact on HAIs** | | | | |
| **Objective 1:** Define diagnostic stewardship and explain connection to hospital epidemiology | | X | X |  |
|  | [Review the SHEA position paper on diagnostic stewardship](https://www.cambridge.org/core/journals/infection-control-and-hospital-epidemiology/article/principles-of-diagnostic-stewardship-a-practical-guide-from-the-society-for-healthcare-epidemiology-of-america-diagnostic-stewardship-task-force/324C90B8F54885D28CC9218433901E0A) |  |  |  |
| **Objective 2:** Illustrate principles of diagnostic stewardship in infection prevention using examples, such as *C. difficile*, blood cultures, and urine cultures, etc | | X | X |  |
|  | Review institutional ordering practices and guidelines on urine cultures, *C. difficile* infection testing, and blood cultures. |  |  |  |
|  | Apply diagnostic stewardship principles to an existing, new, or potential project within your hospital’s IP program. |  |  | X |
| **Objective 3:** Discuss the connections between diagnostic and antimicrobial stewardship in the context of hospital epidemiology/infection prevention (ex. *C. difficile*, urine culturing, etc) | |  |  | X |
|  |  |  |  |  |
| **Antimicrobial stewardship** | | | | |
| **Objective 1:** Illustrate the connections between diagnostic and antimicrobial stewardship in the context of hospital epidemiology/infection prevention (ex. *C. difficile*, urine culturing, etc) | |  | X |  |
|  | Discuss an active diagnostic stewardship project from your facility and how it benefits both IP and antimicrobial stewardship |  |  |  |
|  |  |  |  |  |
| **Microbiology lab** | | | | |
| **Objective 1:** Recognize the relationship between the microbiology laboratory and infection prevention (blood cultures, MDROs, urine cultures, *C. difficile* testing) | |  | X |  |
|  | Meet with local microbiology lab and review policies on blood cultures, MDROs, urine cultures, *C. difficile* testing. |  |  |  |
|  |  |  |  |  |
| **OCCUPATIONAL HEALTH** | | **Core** | **Basic** | **Advanced** |
|  |  |  |  |  |
| **Staff vaccination** | | | | |
| **Objective 1:** Identify core vaccinations recommended for healthcare personnel and describe their importance for infection prevention | | X | X |  |
|  | [Review the Advisory Committee on Immunization Practices (ACIP) recommendations for vaccination of HCP](https://www.cdc.gov/mmwr/preview/mmwrhtml/rr6007a1.htm) |  |  |  |
|  | [Review Hepatitis B vaccination requirements under the OSHA Bloodborne Pathogen Standard](https://www.osha.gov/sites/default/files/publications/bbfact05.pdf) |  |  |  |
| **Objective 2:** Describe the impact of HCP influenza vaccination on influenza-like illness and mortality in long-term care and acute care hospitals | |  | X |  |
| **Objective 3:** Describe strategies to improve vaccination rates among HCP | |  |  | X |
|  | [Review Joint Commission Strategies for Improving Health Care Personnel Influenza Vaccination Rates](https://www.jointcommission.org/-/media/tjc/documents/resources/hai/strategies_-__improving_health_care_personnel_influenza_vaccination_rates.pdf) |  |  |  |
|  |  |  |  |  |
| **Presenteeism** | | | | |
| **Objective 1:** Define presenteeism and describe its potential impact on HAIs | |  | X |  |
| **Objective 2:** Describe strategies to mitigate presenteeism | |  | X |  |
|  | Discuss your facility's challenges with presenteeism with respiratory viruses with IP or occupational medicine |  |  | X |
|  |  |  |  |  |
| **Healthcare worker exposure investigations** | | | | |
| **Objective 1:** Describe principles of evaluating and managing exposures, including blood borne and non-bloodborne pathogens | | X | X |  |
|  | [Review "Occupational Health Update: Approach to Evaluation of Health Care Personnel and Preexposure Prophylaxis"](https://www.ncbi.nlm.nih.gov/pmc/articles/PMC8331250/) |  |  |  |
| **Objective 2:** Identify which body fluids and exposure mechanisms are considered high risk | |  | X |  |
| **Objective 3:** Identify and provide management recommendations for infections in which post-exposure prophylaxis is indicated | |  | X |  |
| **Objective 4:** Distinguish the various postexposure work restrictions for infected or exposed asymptomatic HCP (e.g. *N. meningitidis*, VZV, Measles, Influenza, Norovirus, Pertussis) | |  |  | X |
|  |  |  |  |  |
| **TB screening and latent TB in healthcare workers** | | | | |
| **Objective 1:** Interpret the results of pre-placement TB screening and provide treatment recommendations | |  | X |  |
|  | [Review the American College of Occupational and Environmental Medicine (ACOEM) and National Tuberculosis Controllers Association (NTCA) Joint Task Force recommendations for screening, testing, and treating for TB in U.S. healthcare personnel](https://journals.lww.com/joem/fulltext/2020/07000/tuberculosis_screening,_testing,_and_treatment_of.22.aspx) |  |  |  |
| **Objective 2:** Describe what qualifies as an exposure TB in the healthcare setting and management/follow-up testing | |  | X |  |
|  | Review your facility’s policies/procedures for TB exposure management |  |  |  |
| **Objective 3:** Describe methods for managing borderline IGRAs in HCP | |  |  | X |
|  |  |  |  |  |
| **EMERGENCY PREPAREDNESS** | | **Core** | **Basic** | **Advanced** |
|  |  |  |  |  |
| **Emerging pathogens** | | | | |
| **Objective 1:** Define emerging pathogens, including bioterrorism-related and re-emerging infectious diseases | | X | X |  |
|  | [Review NIAID's list of emerging and re-emerging pathogens](https://www.niaid.nih.gov/research/emerging-infectious-diseases-pathogens) |  |  |  |
| **Objective 2:** Describe syndrome-based isolation/control measures for emerging infectious disease disasters in which the agent is not yet identified | |  |  | X |
|  | [Review APIC's Guide to Infection Prevention in Emergency Medical Services, Section 7: "Bioterrorism and Infectious Disease Emergency Preparedness"](https://nasemso.org/wp-content/uploads/Guide-to-Infection-Prevention-in-EMS-APIC.pdf) |  |  | X |
|  |  |  |  |  |
| **Hospital Incident Command** | | | | |
| **Objective 1:** Recognize the role and importance of the Incident Command System (ICS) and Hospital Incident Command System (HICS) | |  |  | X |
|  | [Review the NIMS course](https://training.fema.gov/is/courseoverview.aspx?code=IS-100.c&lang=en) |  |  |  |
|  | [Review SHEA's guidance on "Outbreak Response and Incident Management"](https://www.cambridge.org/core/journals/infection-control-and-hospital-epidemiology/article/outbreak-response-and-incident-management-shea-guidance-and-resources-for-healthcare-epidemiologists-in-united-states-acutecare-hospitals/8C035426B3C86E075BBB0AFFAE42F0AC) |  |  |  |
|  |  |  |  |  |
| **Collaboration with state and local public health** | | | | |
| **Objective 1:** Describe the purpose and scope of the CDC’s Health Alert Network (HAN) messaging system | | X | X |  |
|  | [Review the most recent updates from the HAN](https://emergency.cdc.gov/han/index.asp) |  |  |  |
| **Objective 2:** Identify community workers requiring infection prevention education during an infectious disease disaster | |  | X |  |
|  | [Review APIC's "Ambulatory Care During Disasters"](https://apic.org/Resource_/TinyMceFileManager/Emergency_Prep/2013_Ambulatory_Care_during_Disasters_FINAL.pdf) |  |  |  |
| **Objective 3:** Describe policy development in response to infection-related events | |  |  | X |
|  | [Review the CDC's public health emergency response guide for state, local, and Tribal health departments](https://emergency.cdc.gov/planning/responseguide.asp) |  |  |  |
|  | Review the development of standard operating procedures for the following: (a) Rapid identification and isolation of suspected cases among patients and workers (b) Safe processes for sample collection and transport (c) Rapid contact tracing (d) Assess requirements for personal protective equipment (PPE) and supplies and develop contingency plans in case of supply shortages |  |  | X |
|  | Assist in policy and response plan development and evaluation for infection-related events (real or simulated), such as bioterrorism or pandemic respiratory pathogens |  |  | X |
|  | Develop or participate in a simulation or table-top exercise to test a local outbreak plan |  |  | X |
|  |  |  |  |  |
| **Principles of pandemic preparedness** | | | | |
| **Objective 1:** Describe the basics of developing a pandemic preparedness plan | |  | X |  |
|  | [Review the CDC's resources for developing national, state, or local pandemic influenza preparedness plans](https://www.cdc.gov/pandemic-flu/php/national-strategy/index.html) |  |  |  |
| **Objective 2:** Recognize the importance of identifying and triaging potentially contagious individuals upon entering a facility, including ambulatory and community facilities | |  | X |  |
|  | Develop a plan for establishing a triage area to identify and separate potentially contagious individuals upon entering a hospital |  |  |  |
|  | [Review APIC's "Ambulatory Care During Disasters"](https://apic.org/Resource_/TinyMceFileManager/Emergency_Prep/2013_Ambulatory_Care_during_Disasters_FINAL.pdf) |  |  |  |
|  | Summarize methods to reduce disease transmission from potentially contagious patients who are triaged to alternative care sites, such as clinics, community-based evacuation shelters, and long-term care facilities. |  |  | X |
| **Objective 3:** Recognize the CMS Emergency Preparedness regulations and requirements to participate in the Medicare or Medicaid program | |  |  | X |
|  | [Complete the CMS Emergency Preparedness Basic Training](https://qsep.cms.gov/pubs/CourseMenu.aspx?cid=0CMSEmPrep_ONL) |  |  |  |
| **Objective 4:** Identify methods to reduce healthcare personnel absenteeism during an infectious disease disaster, including: prioritizing select healthcare personnel vaccination, offering prophylaxis and vaccination to personnel family members, such as EMS, volunteers, etc. | |  |  | X |
|  | Discuss what your facility has done for prior infectious diseases disasters at your facility with infection prevention or other pandemic preparedness team members |  | X |  |
|  |  |  |  |  |
| **HOSPITAL LEADERSHIP AND OPERATIONS** | | **Core** | **Basic** | **Advanced** |
|  |  |  |  |  |
| **Hospital administration structure** | | | | |
| **Objective 1:** Describe the structure of your institution’s IPC/HE program, including how it fits in the institutional organizational chart | |  | X |  |
|  | Review institutional organizational chart and where IP/HE program sits within that |  |  |  |
|  |  |  |  |  |
| **Policy creation** | | | | |
| **Objective 1:** Recognize how IP policies are created and modified with new data | |  | X |  |
|  | Review IP policies and modification/creation process with infection preventionists |  |  |  |
|  |  |  |  |  |
| **Return on investment and paying for IP work** | | | | |
| **Objective 1:** Recognize the importance of making a financial case for IP | |  | X |  |
|  | [Read ICHE article on making a business case](https://www.cambridge.org/core/journals/infection-control-and-hospital-epidemiology/article/raising-standards-while-watching-the-bottom-line-making-a-business-case-for-infection-control/F5B126A74D8B7BDBDC6309D5F6583E05) |  |  |  |
|  | [Review CDC's "Creating a Business Case for Infection Prevention"](https://www.cdc.gov/infectioncontrol/pdf/strive/BC101-508.pdf) |  |  |  |
| **Objective 2:** List the outcomes that can be measured to estimate the cost of an HAI, such as: number of bed-days lost to a case of HAI, length of stay, hospital charges, mortality | |  |  | X |
| **Objective 3:** Apply return on investment (ROI) principles to an existing or potential IP/HE issue | |  |  | X |
|  |  |  |  |  |
| **Quality improvement** | | | | |
| **Objective 1:** Summarize a quality improvement framework (e.g. Lean principles, plan do study act, Six Sigma, DMAIC) and apply it to a potential or existing IP problem | | X | X |  |
|  | Review process for root cause analysis, use local QI frameworks (e.g. Lean principles, plan do study act, Six Sigma, DMAIC). |  |  |  |
| **Objective 2:** Interpret and display HAI metric data, such as run charts and data dashboards | |  | X |  |
|  | Familiarize with institutional data tools and dashboards for HAI data |  |  |  |
| **Objective 3:** Interpret HAI metric data using SPC charts | |  |  | X |
| **Objective 4:** Recognize the role of the SQUIRE guidelines in publishing QI work | |  |  | X |
|  |  |  |  |  |
| **COMMUNICATING IP WORK** | | **Core** | **Basic** | **Advanced** |
|  |  |  |  |  |
| **Publishing IP work** | | | | |
| **Objective 1:** Describe the benefits and challenges of publishing infection prevention research | |  | X |  |
|  | Identify a research mentor and work on a clinical learning and/or research project |  |  |  |
| **Objective 2:** Participate in infection prevention research with the goal of manuscript publication or presentation (oral or poster) at a local, regional, or national meeting | |  |  | X |
|  |  |  |  |  |
| **Educating on IP topics** | | | | |
| **Objective 1:** Understand the basics of creating and delivering an education session for trainees or hospital staff on an IP topic | | X | X |  |
|  | Review methods and tools for effective delivery of education, such as: didactic lectures, small-group case-based discussions, one-on-one instruction, simulation, unit-level training, role-play, hands-on skill training, and computer-based modules |  |  |  |
|  | Join an infection preventionist for an educational intervention at your facility (huddle, new employee orientation, etc) |  |  |  |
|  | Use presentation tools to develop effective educational materials targeting specifical healthcare personnel, such as trainees, clinical staff, nonclinical staff, etc, and if feasible, provide that education |  |  | X |
|  |  |  |  |  |
| **Internal and external communication** | | | | |
| **Objective 1:** List available tools for communicating with patients and staff and examine the benefits and challenges of these common tools, such as: SBAR, debriefing, videoconferencing, digital shared file storage, and smartphone chat groups | |  | X |  |
|  | Review techniques to communicate IP updates, such as: SBAR, debriefing, videoconferencing, digital shared file storage, and smartphone chat groups |  |  |  |
|  | [Review the CDC’s Infection Control section on communication and collaboration among healthcare personnel](https://www.cdc.gov/infection-control/hcp/healthcare-personnel-infrastructure-routine-practices/communication-collaboration.html#:~:text=OHS%20staff%20maintain%20effective%20communication%20pathways%20with%20a,safety%20and%20the%20delivery%20of%20occupational%20IPC%20services.) |  |  | X |
|  |  |  |  |  |
| **Data visualization** | | | | |
| **Objective 1:** Recognize the importance of data visualization strategies in improving data communication | |  |  | X |
|  | [Review the CDC’s 2018 “Health Communication Playbook” sections regarding images and graphics](https://vaccineresourcehub.org/resource/toolkit-health-communications-playbook-creating-effective-materials) |  |  |  |
|  | [Review ICHE primer on data visualization](https://www.cambridge.org/core/journals/infection-control-and-hospital-epidemiology/article/primer-on-data-visualization-in-infection-prevention-and-antimicrobial-stewardship/3E4B96F87A53EBEBA5E18F79E80AFCF1) |  |  |  |
|  | Using the CDC NHSN data for your institution and a visualization software (such as Tableau), create a graphics display for frontline staff |  |  |  |
|  |  |  |  |  |
| **Social media** | | | | |
| **Objective 1:** Identify how common social media tools can be used in infection prevention communication | |  | X |  |
|  | Review social media tools, such as: podcasts, videos, websites (such as X/Twitter, Facebook), blogs commonly used in IP communication and review one example of effective use in IP practice (example – your institution’s social media, faculty at your institution) |  |  |  |
|  | Review the CDC’s “The Health Communicator’s Social Media Toolkit” |  |  |  |
|  | [Review the article "Use of “Social Media”—an Option for Spreading Awareness in Infection Prevention"](https://pubmed.ncbi.nlm.nih.gov/33519303/) |  |  | X |
|  | Choose a social media communication strategy and create a table reviewing the inputs, activities, outputs, and short- and long-term outcomes for that strategy |  |  | X |
| **Objective 2:** Discuss the risks associated with social media use | |  |  | X |
|  |  |  |  |  |
| **Media training** | | | | |
| **Objective 1:** Recognize basic media communication skills and strategies for effective IP communication | | X | X |  |
|  | [Review the CDC’s 2018 “Health Communication Playbook”, including communication strategies, consumer communication, media and press communication, and professional communication](https://vaccineresourcehub.org/resource/toolkit-health-communications-playbook-creating-effective-materials) |  |  |  |
|  | Discuss a local example of where media commutation of critical for information dissemination in your facility with the infection prevention team |  |  |  |
|  | Meet with local public affairs team to discuss key effectors of effective media communication |  |  | X |
