# Supplementary material for: Society for Healthcare Epidemiology of America (SHEA) infectious diseases fellow infection prevention and control and healthcare epidemiology curriculum
Source: Infect Control Hosp Epidemiol. 2025 Jun 3;46(8):773–82. doi: 10.1017/ice.2025.85 (PMC12483618; doi:10.1017/ice.2025.85)
Supplement: Martin et al. supplementary material 2 — Martin et al. supplementary material [file S0899823X25000856sup002.docx]

**Supplementary Table 1: IPC/HE Curriculum Objectives.**

| **SURVEILLANCE AND REPORTING** | **Core** | **Basic** | **Advanced** |
| --- | --- | --- | --- |
| **NHSN Definitions (CAUTI, CLABSI, CDI, MRSA, VAE, SSI)** | | | |
| **Objective 1:** Identify current NHSN definitions for the various HAIs | X | X |  |
| **Objective 2:** Differentiate between surveillance definitions and clinical definitions for HAIs |  | X |  |
| **Objective 3:** Explain advantages and disadvantages of NHSN surveillance definitions, including over capture vs under capture, LabID events, etc |  |  | X |
| **HAI Performance Metrics** | | | |
| **Objective 1:** Compare the different metrics used in HAI measures (i.e. rates vs SIRS) | X | X |  |
| **Objective 2:** Identify organizations to which HAI metrics are reported, including reporting sites such as Hospital Compare, Leapfrog, etc. |  |  | X |
| **Objective 3:** Describe the impact of HAI rates on insurance reimbursements, including Value Based Purchasing |  |  | X |
| **State and local county requirements for reporting** | | | |
| **Objective 1:** Identify HAIs that are reportable to local and state health departments, as defined by regional legislation |  |  | X |
| **CLUSTER DETECTION, INVESTIGATION AND RESOLUTION** | **Core** | **Basic** | **Advanced** |
| **Cluster/Outbreak Investigation** | | | |
| **Objective 1:** Define an epidemiologically significant cluster and recognize differences in cluster detection thresholds among pathogens | X | X |  |
| **Objective 2:** Describe the steps to conduct an outbreak investigation, including case definition, line list, epidemic curves, communication strategies, and public health notification |  | X |  |
| **Objective 3:** Describe different techniques used in outbreak investigations to confirm transmission/genetic relatedness including organism identification, sensitivities, and whole genome sequencing. |  |  | X |
| **Objective 4:** Demonstrate effective communication to hospital leadership, risk management, clinical staff, patients/families, etc |  |  | X |
| **Objective 5:** Differentiate between an outbreak and a pseudo-outbreak |  | X |  |
| **Objective 6:** Describe how to approach an outbreak investigation when no clear source is identified |  |  | X |
| **Patient Exposure investigation** | | | |
| **Objective 1:** Explain the key elements of the patient exposure investigation including identifying patients potentially exposed; incubation period; post-exposure measures including prophylaxis, vaccination, monitoring, post discharge isolation, hospital reporting | X | X |  |
| **Surveillance and monitoring** | | | |
| **Objective 1:** Understand different methods for cluster detection/monitoring, including reactive (following positive clinical cultures) vs proactive assessment (active surveillance) that is used in your facility |  | X |  |
| **PATHOGEN TRANSMISSION AND TRANSMISSION INTERRUPTION** | **Core** | **Basic** | **Advanced** |
| **Modes of Transmission** | | | |
| **Objective 1:** Describe ways in which patients acquire hospital-acquired infections (patient to patient, from hospital/environment, & from their own flora) and identify examples for each mode of transmission |  | X |  |
| **Transmission-based precautions** | | | |
| **Objective 1:** Define and list transmission-based precautions used in healthcare | X | X |  |
| **Objective 2**: Understand how each transmission-based precautions decreases the risk of pathogen transmission |  | X |  |
| **Objective 3:** Describe methods for auditing transmission-based precautions |  | X |  |
| **Objective 4:** Recognize evidence-based practices that can increase compliance with transmission-based precautions |  |  | X |
| **Objective 5:** Appreciate differences in isolation and discontinuation practices between institutions |  |  | X |
| **Tuberculosis prevention** | | | |
| **Objective 1:** Identify strategies used by healthcare facilities to reduce the risk of hospital acquired TB for patients and staff |  | X |  |
| **Standard precautions** | | | |
| **Objective 1:** Explain how standard precautions are used to protect both staff and patients from pathogen transmission | X | X |  |
| **Objective 2:** Understand the role of hand hygiene in infection prevention | X | X |  |
| **Objective 3:** Appreciate the basics of injection safety |  | X |  |
| **Objective 4:** Appreciate the basics of respiratory precautions |  | X |  |
| **Special populations** | | | |
| **Objective 1:** Describe additional strategies used to reduce transmission of pathogens in special populations or specialized locations (BMT, Solid organ transplant, Burn units, dialysis units, NICU/PICU, Ambulatory, L&D, long term care, ORs, and resource limited settings) |  |  | X |
| **Prevention strategies for CAUTI, CLABSI, CDI, MRSA, MDRO, SSI, etc including bundles** | | | |
| **Objective 1:** Recognize effective strategies to reduce HAIs and/or device associated infections | X | X |  |
| **Objective 2:** Describe effective strategies to reduce CAUTI |  | X |  |
| **Objective 3:** Describe effective strategies to reduce CLABSI |  | X |  |
| **Objective 4:** Describe effective strategies to reduce *C. difficile* |  | X |  |
| **Objective 5:** Describe effective strategies to reduce MRSA |  | X |  |
| **Objective 6:** Describe effective strategies to reduce SSI |  | X |  |
| **Objective 7:** Describe effective strategies to reduce VAE |  | X |  |
| **ENVIRONMENT OF CARE** | **Core** | **Basic** | **Advanced** |
| **Construction** | | | |
| **Objective 1:** Recognize pathogens associated with specific water and construction activities (Aspergillus and molds, waterborne pathogens) | X | X |  |
| **Objective 2:** Identify construction related activities associated with HAIs |  | X |  |
| **Objective 3:** Describe mitigation strategies for construction-associated HAIs |  |  | X |
| **Objective 4:** Define the purpose and general elements of an Infection Control Risk Assessment |  |  | X |
| **Water Management** | | | |
| **Objective 1:** Identify reservoirs for common waterborne pathogens (Legionella, Pseudomonas, other Gram negatives, mycobacteria/NTM) | X | X |  |
| **Objective 2:** Describe strategies to interrupt the transmission of water borne pathogens, including having a water management plan |  | X |  |
| **Objective 3:** Review the potential infectious risks associated with bathroom fixtures, such as toilet and sinks |  |  | X |
| **Objective 4:** Recognize unique infection prevention requirements and strategies in hemodialysis |  | X |  |
| **Air Quality** | | | |
| **Objective 1:** Explain the role of air handling and pressure differentials to reduce the risk of pathogen transmission |  | X |  |
| **Objective 2:** Compare the air handling and pressure requirements for different areas of the healthcare facility | |  | X |
| **Environmental Cleaning, Disinfection, and Sterilization** | | | |
| **Objective 1:** Explain the role of environmental and reusable medical device cleaning in transmission disruption |  | X |  |
| **Objective 2:** Describe how to select an appropriate cleaning agent for a device (sporicidal agents, indications for use) |  | X |  |
| **Objective 3:** Discuss the role of UV disinfection and other no touch cleaning strategies, including indications, benefits, and limitations |  |  | X |
| **Objective 4:** Compare methods of low-level disinfection, high-level disinfection, and sterilization for reusable medical devices | X | X |  |
| **Objective 5:** Review benefits and limitations of common methods to assess the effectiveness of cleaning (direct practice observation, fluorescent markers, ATP bioluminescence testing, environmental cultures, etc.) |  |  | X |
| **DIAGNOSTIC STEWARDSHIP** | **Core** | **Basic** | **Advanced** |
| **Test selection, test characteristics, and impact on HAIs** | | | |
| **Objective 1:** Define diagnostic stewardship and explain connection to hospital epidemiology. | X | X |  |
| **Objective 2:** Illustrate principles of diagnostic stewardship in infection prevention using examples, such as CDI, blood and urine cultures, etc | X | X |  |
| **Objective 3:** Discuss the connections between diagnostic and antimicrobial stewardship in the context of hospital epidemiology/infection prevention (ex. *C. difficile*, urine culturing, etc) |  |  | X |
| **Antimicrobial stewardship** | | | |
| **Objective 1:** Illustrate the connections between diagnostic and antimicrobial stewardship in the context of hospital epidemiology/infection prevention (ex. *C. difficile*, urine culturing, etc) |  | X |  |
| **Microbiology lab** | | | |
| **Objective 1:** Recognize the relationship between the microbiology laboratory and infection prevention (blood cultures, MDROs, urine cultures, CDI.) |  | X |  |
| **OCCUPATIONAL HEALTH** | **Core** | **Basic** | **Advanced** |
| **Staff vaccination** | | | |
| **Objective 1:** Identify core vaccinations recommended for healthcare personnel and describe their importance for infection prevention | X | X |  |
| **Objective 2:** Describe the impact of HCP influenza vaccination on influenza-like illness and mortality in long-term care and acute care hospitals |  | X |  |
| **Objective 3:** Describe strategies to improve vaccination rates among HCP |  |  | X |
| **Presenteeism** | | | |
| **Objective 1:** Define presenteeism and describe its potential impact on HAIs |  | X |  |
| **Objective 2:** Describe strategies to mitigate presenteeism |  | X |  |
| **Healthcare worker exposure investigations** | | | |
| **Objective 1:** Describe principles of evaluating and managing exposures, including blood borne and non-bloodborne pathogens | X | X |  |
| **Objective 2:** Identify which body fluids and exposure mechanisms are considered high risk |  | X |  |
| **Objective 3:** Identify and provide management recommendations for infections in which post-exposure prophylaxis is indicated |  | X |  |
| **Objective 4:** Distinguish the various postexposure work restrictions for infected or exposed asymptomatic HCP (e.g. *N. meningitidis*, VZV, Measles, Influenza, Norovirus, Pertussis) |  |  | X |
| **TB screening and latent TB in healthcare workers** | | | |
| **Objective 1:** Interpret the results of pre-placement TB screening and provide treatment recommendations |  | X |  |
| **Objective 2:** Describe what qualifies as an exposure TB in the healthcare setting and management/follow-up testing |  | X |  |
| **Objective 3:** Describe methods for managing borderline IGRAs in HCP |  |  | X |
| **EMERGENCY PREPAREDNESS** | **Core** | **Basic** | **Advanced** |
| **Emerging pathogens** | | | |
| **Objective 1:** Define emerging pathogens, including bioterrorism-related and re-emerging infectious diseases | X | X |  |
| **Objective 2:** Describe syndrome-based isolation/control measures for emerging infectious disease disasters in which the agent is not yet identified |  |  | X |
| **Hospital Incident Command** | | | |
| **Objective 1:** Recognize the role and importance of the Incident Command System (ICS) and Hospital Incident Command System (HICS) |  |  | X |
| **Collaboration with state and local public health** | | | |
| **Objective 1:** Describe the purpose and scope of the CDC’s Health Alert Network (HAN) messaging system | X | X |  |
| **Objective 2:** Identify community workers requiring infection prevention education during an infectious disease disaster |  | X |  |
| **Objective 3:** Describe policy development in response to infection-related events |  |  | X |
| **Principles of pandemic preparedness** | | | |
| **Objective 1:** Describe the basics of developing a pandemic preparedness plan |  | X |  |
| **Objective 2:** Recognize the importance of identifying and triaging potentially contagious individuals upon entering a facility, including ambulatory and community facilities |  | X |  |
| **Objective 3:** Recognize the CMS Emergency Preparedness regulations and requirements to participate in the Medicare or Medicaid program |  |  | X |
| **Objective 4:** Identify methods to reduce healthcare personnel absenteeism during an infectious disease disaster, including: prioritizing select healthcare personnel vaccination, offering prophylaxis and vaccination to personnel family members, such as EMS, volunteers, etc. |  |  | X |
| **HOSPITAL LEADERSHIP AND OPERATIONS** | **Core** | **Basic** | **Advanced** |
| **Hospital administration structure** | | | |
| **Objective 1:** Describe the structure of your institution’s IPC/HE program, including how it fits in the institutional organizational chart |  | X |  |
| **Policy creation** | | | |
| **Objective 1:** Recognize how IP policies are created and modified with new data |  | X |  |
| **Return on investment and paying for IP work** | | | |
| **Objective 1:** Recognize the importance of making a financial case for IP |  | X |  |
| **Objective 2:** List the outcomes that can be measured to estimate the cost of an HAI, such as: number of bed-days lost to a case of HAI, length of stay, hospital charges, mortality |  |  | X |
| **Objective 3:** Apply return on investment (ROI) principles to an existing or potential IP/HE issue |  |  | X |
| **Quality improvement** | | | |
| **Objective 1:** Summarize a quality improvement framework (e.g. Lean principles, plan do study act, Six Sigma, DMAIC) and apply it to a potential or existing IP problem | X | X |  |
| **Objective 2:** Interpret and display HAI metric data, such as run charts and data dashboards |  | X |  |
| **Objective 3:** Interpret HAI metric data using SPC charts |  |  | X |
| **Objective 4:** Recognize the role of the SQUIRE guidelines in publishing QI work |  |  | X |
| **COMMUNICATING IP WORK** | **Core** | **Basic** | **Advanced** |
| **Publishing IP work** | | | |
| **Objective 1:** Describe the benefits and challenges of publishing infection prevention research |  | X |  |
| **Objective 2:** Participate in infection prevention research with the goal of manuscript publication or presentation (oral or poster) at a local, regional, or national meeting |  |  | X |
| **Educating on IP topics** | | | |
| **Objective 1:** Understand the basics of creating and delivering an education session for trainees or hospital staff on an IP topic | X | X |  |
| **Internal and external communication** | | | |
| **Objective 1:** List available tools for communicating with patients and staff and examine the benefits and challenges of these common tools, such as: SBAR, debriefing, videoconferencing, digital shared file storage, and smartphone chat groups |  | X |  |
| **Data visualization** | | | |
| **Objective 1:** Recognize the importance of data visualization strategies in improving data communication. |  |  | X |
| **Social media** | | | |
| **Objective 1:** Identify how common social media tools can be used in infection prevention communication |  | X |  |
| **Objective 2:** Discuss the risks associated with social media use |  |  | X |
| **Media training** | | | |
| **Objective 1:** Recognize basic media communication skills and strategies for effective IP communication | X | X |  |
